# Puerperal Fever

**Published:** 1853-10

**Authors:** 


*Puerperal Fever.*—MM. Paul Dubois and Grissolle have employed the tincture of aconite in this affection in three cases; two of the patients died after a much longer period than puerperal fever usually requires to arrive at its fatal termination; the autopsies revealed numerous abscesses, pus in the uterine sinuses, and the other lesions of puerperal fever. The third patient recovered after presenting all the symptoms of purulent infection, and left M. Dubois' ward perfectly well. M. Tessier has recommended aconite in large doses in purulent absorption. Clinical experience can alone decide whether the remedy is worthy of confidence.—*Revue de Therap.*, from *Vir. Med. and Surg. Jour.*

